# Many-to-Many Multicast Routing Schemes under a Fixed Topology

**DOI:** 10.1155/2013/718152

**Published:** 2013-03-25

**Authors:** Wei Ding, Hongfa Wang, Xuerui Wei

**Affiliations:** ^1^Zhejiang Water Conservancy and Hydropower College, Hangzhou, Zhejiang 310018, China; ^2^Department of Mathematics, Shaoxing University, Shaoxing, Zhejiang 312000, China

## Abstract

Many-to-many multicast routing can be extensively applied in computer or communication networks supporting various continuous multimedia applications. The paper focuses on the case where all users share a common communication channel while each user is both a sender and a receiver of messages in multicasting as well as an end user. In this case, the multicast tree appears as a terminal Steiner tree (TeST). The problem of finding a TeST with a quality-of-service (QoS) optimization is frequently NP-hard. However, we discover that it is a good idea to find a many-to-many multicast tree with QoS optimization under a fixed topology. In this paper, we are concerned with three kinds of QoS optimization objectives of multicast tree, that is, the minimum cost, minimum diameter, and maximum reliability. All of three optimization problems are distributed into two types, the centralized and decentralized version. This paper uses the dynamic programming method to devise an exact algorithm, respectively, for the centralized and decentralized versions of each optimization problem.

## 1. Introduction

Multicast routing has been increasingly used in computer or communication networks supporting various multimedia applications, such as real-time audio and video conferences, entertainment, and distance learning, [1, 2]. Multicast routing is known as a *multicast tree* [3], which can reduce the usage of network resource, such as network cost and bandwidth. Multicast tree can be reduced to be a *Steiner tree* in mathematics [4]. 

In a real world, a large number of continuous multimedia applications drive the consumers to advance their *quality of service* (QoS) requirements [2, 5, 6] (e.g., cost, delay, and bandwidth). As we all know, the minimum cost multicast tree (Steiner tree) problem is NP-hard [7] and the minimum diameter multicast tree (Steiner tree) problem is polynomial solvable [8]. Furthermore, the multicast tree problem with additional QoS requirements is frequently harder to solve. For example, in past decade, a number of heuristics [9, 10] and distributed algorithms [11, 12] have been devised for finding a minimum cost delay-constrained multicast tree. Surprisingly, the *fixed topology* version of this problem, in which the configuration of multicast tree is given in advance with a tree topology, is easier than the classic version. Wang and Jia [13] designed a pseudo-polynomial-time algorithm, and Xue and Xiao [14] devised a full polynomial time approximation scheme. Not only that, we believe the idea of under a fixed topology will play an important role in exploring a desired multicast routing with a variety of QoS requirements.

In many practical settings, each destination is a *terminal*. A terminal means an end user, who takes charge of receiving and sending data but not branching them, that is, it can not serve as a *relay node* in charge of copying and branching data to other terminals. In a word, a terminal is a source, a receiver, or both. For instance, a member in video conference not only receives all the others' real-time images but also sends its real-time images to all the others. In fact, this is a type of general paradigm of *many-to-many* multicast routing. Provided that all terminals share a common communication channel, one many-to-many multicast tree can be reduced to a *terminal Steiner tree* (TeST) [15]. The minimum cost TeST problem is known to be NP-hard [15] and the minimum diameter TeST problem is polynomial solvable [8]. Many-to- many multicast tree includes two types, the *centralized* and *decentralized*. In the former, a network node with a bootstrap serves as a server in charge of receiving a terminal's data and then copying and branching them to all the other terminals by using multicast, resulting in a centralized multicast tree, essentially a rooted TeST (with the root at the server node). The centralized multicast tree problem is in fact *one-to-many* multicast tree problem [13, 14, 16]. In the latter, a terminal sends its data directly to all the others using a common channel, resulting in a decentralized one, an unrooted TeST in essence. 

To the best of our knowledge, there have been a number of studies on the unrestricted many-to-many multicast tree mentioned above (terminal Steiner tree) [8, 15, 17–19]; however few studies on the QoS restricted version [16] due to its vast difficulty. Fortunately, we discover that the idea of under a fixed topology could provide us with a new way of studying the restricted version. In this paper, we will study the unrestricted many-to-many multicast tree problem with the objective of cost minimized or delay minimized and/or reliability maximized under a fixed topology, respectively. The approaches and results presented in the rest of the paper will contribute to study the many-to-many multicast tree with QoS restrictions under a fixed topology in the recent future. 

The rest of this paper is organized as follows. In Section 2, we introduce the architecture of many-to-many multicast tree under a fixed topology. In Section 3, we make preliminaries, including defining three kinds of metrics of QoS of multicast tree, three QoS optimization problems, and two Euclidean graphs. We present an exact algorithm, respectively, for the centralized and decentralized version of the minimum cost problem in Section 4, the minimum delay problem in Section 5, and maximum reliability problem in Section 6. In Section 7, we give an example to illustrate all the algorithms. In Section 8, we conclude the paper with some research topics. 

## 2. Architecture of Many-to-Many Multicast Tree under a Fixed Topology

A computer or communication network is frequently modeled as an undirected graph [20]. Let *G* = (*V*, *E*, *ω*) be an undirected edge-weighted graph with a subset *S* ⊂ *V* of terminals and *T* = (*U*, *F*) be a sample TeST. Here, *V* and *E* denote the node set and edge set of *G*, *U* and *F* denote the node set and edge set of *T*, and *ω*(*e*) denotes the weight on *e* for every *e* ∈ *E*. For any edge *f* = {*u*_*i*_, *u*_*j*_} ∈ *F*, we say that *f* is realized in *G* once its two endpoints *u*_*i*_ and *u*_*j*_ are mapped to two different nodes *v*_*i*_ and *v*_*j*_ in *G*. Note that *u*_*i*_ and *u*_*j*_ are not allowed to be mapped to a same node of *G*. In essence, *f* is mapped to a simple path in *G* connecting *v*_*i*_ and *v*_*j*_, often a so-called optimal path with respect to some optimization objective, for example, a shortest path if *ω*(*e*) represents the length of *e*. Also, we use *R*(*f*) to denote a realization of *f* in *G*, and use π*[*v*_*i*_, *v*_*j*_] to denote an optimal path in *G* between *v*_*i*_ and *v*_*j*_ for any a pair of nodes *v*_*i*_ and *v*_*j*_. 

Given any two different nodes *x* and *y* of *T*, there is a unique path in *T* between them. Specially, if *x* and *y* are both leaves, we call the path a *leaf-to-leaf path* of *T*, denoted as *L*[*x*, *y*]. In addition, the path in *T* between the root node *r* and a leaf *x* is called a *root-to-leaf path* of *T*, denoted as *C*[*r*, *x*]. Let *R*(*x*, *y*) denote a realization of *L*[*x*, *y*] in *G* and *R*(*r*, *x*) a realization of *C*[*r*, *x*]. It is clear that *R*(*x*, *y*) (resp. *R*(*r*, *x*)) is composed of all the realizations of edges on *L*[*x*, *y*] (resp. *C*[*r*, *x*]) respectively. Furthermore, all the realizations of the edges on *T* form a realization of *T* in *G*, denoted as *R*(*T*). A realization of *T* in *G* forms a many-to-may multicast tree under a fixed topology *T* in *G* essentially. 

**Figure 1:**
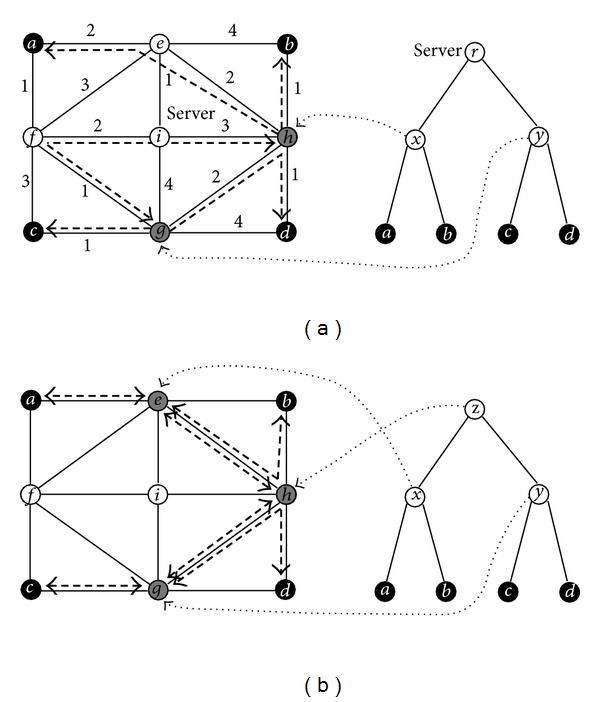
Ignore the dashed lines on the left top subfigure to obtain a sample graph where the number on each edge represents its length. The right top subfigure is a sample rooted TeST and the right bottom is a sample unrooted TeST. An example centralized many-to-many multicast tree under a fixed topology is distinguished by dashed lines on the left top subfigure and an example decentralized one is distinguished on the left bottom subfigure.

In this paper, we are concerned with the centralized and decentralized multicast trees under a fixed topology, namely, the realization of a given rooted and unrooted TeST topology; see Figure 1. On the top subfigure, the left graph shows a network with each edge having a weight denoting its length and the right graph shows a given rooted TeST topology. A realization of the rooted TeST in the network is distinguished by dashed edges. Considering that every leaf of the TeST is required to be mapped to a fixed node (terminal) and its root is required to be mapped to the server node, the essential work of realizing the TeST in the network is to map all the nonleaves except the root of the TeST to some nonterminals. If we arrange all the nonleaves in the order of from the 2nd level to top and from left to right on a level and label them by numbers 1,2,…, |*U*∖*S*| in sequence, we can denote all the nonleaves by an ordering (*x*, *y*, *r*), and then use another ordering (*h*, *g*, *i*) to record all the selected nonterminals in the graph. Likewise, on the bottom subfigure, the left shows a same network as above and the right shows an unrooted TeST topology. A realization of the TeST is distinguished by dashed edges. Since each leaf of the TeST is required to be mapped to a fixed node (terminal), the essence of realizing the TeST in the network is to map all the nonleaves of the TeST to some nonterminals. We can transform an unrooted tree into a rooted tree by assigning any a nonleaf as the root, and similarly use (*x*, *y*, *z*) to denote all the nonleaves and (*e*, *g*, *h*) to record all the selected nonterminals in the graph. In general, we can use an ordering (*u*_1_,…, *u*_|*U*∖*S*|_), *u*_*i*_ ∈ *U*∖*S* to denote all the nonleaves of a given TeST and use another ordering (*v*_1_,…, *v*_|*U*∖*S*|_), *v*_*i*_ ∈ *V*∖*S* to record all the selected nonterminal nodes in sequence, that is, a multicast tree under a fixed topology. In this paper, we always use |·| to denote the cardinality of a set. 

Let *T* be a rooted tree, *T*_*u*_ the subtree of *T* rooted at *u*, and *R*(*T*_*u*_) a realization of *T*_*u*_. When *u* is a nonleaf node, we let Δ(*u*) be the set of all the children of *u*, and then immediately obtain that

(1) $$\begin{array}{c} \sum_{u\in U\setminus S}^{}\left|\Delta \left(u\right)\right|=\left|F\right|. \end{array}$$

## 3. Fundamental Preliminaries

In the section, we make some fundamental preliminaries, which will help us to analyze the problems and understand the algorithms proposed in the following.

### 3.1. Metrics

First of all, we define three kinds of metrics of QoS of tree, including the* cost, delay*,and *reliability* of tree. 

#### 3.1.1. Cost

We are given an undirected graph *G* = (*V*, *E*, *c*) where *c*(*e*) represents the cost on *e* for every *c*(·). Let *π* be a path in *G*. The cost of *π* is equal to the sum of all the costs of edges on *π*, that is, *c*(*π*) = ∑_*e*∈*π*_ *c*(*e*). Hence, the cost of edge realization *R*(*f*) of *f* = {*u*_*i*_, *u*_*j*_} in which *u*_*i*_ and *u*_*j*_ are mapped to two different nodes *v*_*i*_ and *v*_*j*_ in *G*, respectively, is denoted as *c*(π*[*v*_*i*_, *v*_*j*_]) where π*[*v*_*i*_, *v*_*j*_] is a shortest path in *G* between *v*_*i*_ and *v*_*j*_. 

The *cost* of *R*(*T*) is defined as the sum of all the costs of edge realizations of *T*, that is,

(2) $$\begin{array}{c} c\left(R\left(T\right)\right)=\sum_{\{u_{i},u_{j}\}\in F}^{}c\left(\pi^{*}\left[v_{i},v_{j}\right]\right). \end{array}$$

Let *R* ^*c*^(*T*) denote a minimum cost realization of *T*. Thus,

(3) $$\begin{array}{c} c\left(R^{c}\left(T\right)\right)=\underset{R\left(T\right)}{\min}\sum_{\{u_{i},u_{j}\}\in F}^{}c\left(\pi^{*}\left[v_{i},v_{j}\right]\right). \end{array}$$

#### 3.1.2. Delay

We are given an undirected graph *G* = (*V*, *E*, *d*) where every edge *e* ∈ *E* has a weight *d*(*e*) representing the delay on *e*. The delay of *π* is equal to the sum of all the delays of edges on *π*, that is, *d*(*π*) = ∑_*e*∈*π*_ *d*(*e*). Then, the delay of edge realization *R*(*f*) is denoted as *d*(π*[*v*_1_, *v*_2_]) in which π*[*v*_*i*_, *v*_*j*_] is a minimum delay path in *G* between *v*_*i*_ and *v*_*j*_. 

The delay of *R*(*x*, *y*) is equal to the sum of all the delays of edge realizations of *L*[*x*, *y*], that is,

(4) $$\begin{array}{c} d\left(R\left(x,y\right)\right)=\sum_{\left\{u_{i},u_{j}\}\in L[x,y\right]}d\left(\pi^{*}\left[v_{i},v_{j}\right]\right). \end{array}$$

Similarly, we have

(5) $$\begin{array}{c} d\left(R\left(r,x\right)\right)=\sum_{\{u_{i},u_{j}\}\in C\left[r,x\right]}^{}d\left(\pi^{*}\left[v_{i},v_{j}\right]\right). \end{array}$$

The maximum delay of leaf-to-leaf path realization of *T* is called the *diameter* of *R*(*T*), that is,

(6) $$\begin{array}{c} \mathrm{diam}\left(R\left(T\right)\right)=\underset{\forall x,y\in S,x\neq y}{\max}d\left(R\left(x,y\right)\right). \end{array}$$

Let *R* ^*d*^(*T*) denote a minimum delay realization of *T*. So,

(7) $$\begin{array}{c} \mathrm{diam}\left(R^{d}\left(T\right)\right)=\underset{R\left(T\right)}{\min}\;\;\underset{\forall x,y\in S,x\neq y}{\max}d\left(R\left(x,y\right)\right). \end{array}$$

The maximum delay of root-to-leaf path realization of *T* is called the *radius* of *R*(*T*), that is,

(8) $$\begin{array}{c} \text{radi}\left(R\left(T\right)\right)=\underset{\forall x\in S}{\max}\;d\left(R\left(r,x\right)\right). \end{array}$$

Likewise,

(9) $$\begin{array}{c} \text{radi}\left(R^{d}\left(T\right)\right)=\underset{R\left(T\right)}{\min}\;\underset{\forall x\in S}{\max}\;d\left(R\left(r,x\right)\right). \end{array}$$

Note that realizations with a minimum diameter or radius are both denoted by *R* ^*d*^(*T*). We can differentiate according to the context where it appears.

#### 3.1.3. Reliability

We are given an undirected graph *G* = (*V*, *E*, *p*) where every edge *e* ∈ *E* has an independent working probability *p*(*e*) while all the nodes are immune to failures. The working probability of *π* is equal to the product of all the working probabilities of edges on *π*, that is, *p*(*π*) = ∏_*e*∈*π*_ *p*(*e*). Then, the working probability of edge realization *R*(*f*) is denoted as *p*(π*[*v*_*i*_, *v*_*j*_]) where π*[*v*_*i*_, *v*_*j*_] is a maximum reliability path in *G* between *v*_*i*_ and *v*_*j*_. 

The working probability of *R*(*x*, *y*), named its *reliability*, is equal to the product of all the working probabilities of edge realizations of *L*[*x*, *y*], that is,

(10) $$\begin{array}{c} p\left(R\left(x,y\right)\right)=\prod_{\{u_{i},u_{j}\}\in L\left[x,y\right]}p\left(\pi^{*}\left[v_{i},v_{j}\right]\right). \end{array}$$

Likewise,

(11) $$\begin{array}{c} p\left(R\left(r,x\right)\right)=\prod_{\{u_{i},u_{j}\}\in C\left[r,x\right]}p\left(\pi^{*}\left[v_{i},v_{j}\right]\right). \end{array}$$

The minimum reliability of leaf-to-leaf path realization of *T* is called the *diameter reliability* of *R*(*T*), that is,

(12) $$\begin{array}{c} \text{dia}p\left(R\left(T\right)\right)=\underset{\forall x,y\in S,x\neq y}{\min}p\left(R\left(x,y\right)\right). \end{array}$$

Let *R* ^*r*^(*T*) denote a maximum reliability realization of *T*. Thus,

(13) $$\begin{array}{c} \text{dia}p\left(R^{r}\left(T\right)\right)=\underset{R\left(T\right)}{\max}\;\;\underset{\forall x,y\in S,x\neq y}{\min}p\left(R\left(x,y\right)\right). \end{array}$$

The minimum reliability of root-to-leaf path realization of *T* is called the *radius reliability* of *R*(*T*), that is,

(14) $$\begin{array}{c} \text{rad}p\left(R\left(T\right)\right)=\underset{\forall x\in S}{\min}p\left(R\left(r,x\right)\right). \end{array}$$

Similarly,

(15) $$\begin{array}{c} \text{rad}p\left(R^{r}\left(T\right)\right)=\underset{R\left(T\right)}{\max}\;\;\underset{\forall x\in S}{\min}\;p\left(R\left(r,x\right)\right). \end{array}$$

Note that realizations with a maximum diameter or radius reliability are both denoted by *R* ^*r*^(*T*). We can differentiate according to the context where it appears.

### 3.2. Problem Definitions

The focus of this paper is three optimization problems of many-to-many multicast tree under a fixed topology from the perspective of three metrics above, which are formally defined as follows.

First of all, we use an INPUT (abbreviated to *I*) to simplify the statements of problems: an undirected graph *G* = (*V*, *E*), a subset *S* ⊂ *V* of terminals, and a sample TeST topology as *T* = (*U*, *F*) (see Figure 1). Let

(16) $$\begin{array}{c} \left|V\right|=n,\;\left|E\right|=m,\;\left|U\right|=\alpha ,\;\left|F\right|=\beta ,\;\left|S\right|=\lambda . \end{array}$$

Moreover, we define $\bar{S}=S\cup \left\{r\right\}$.

**Problem 1**

Given an INPUT with every edge *e* ∈ *E* having a nonnegative weight *c*(*e*) ≥ 0 representing the cost on *e*, the *minimum cost many-to-many multicast tree under a fixed TeST topology problem *(MCMP) aims to find an ordering (*v*_1_,…,*v*_*m*−*k*_)*, *v*_*i*_ ∈ *V*∖*S* with a minimum cost.

**Problem 2**

Given an INPUT with every edge *e* ∈ *E* having a positive weight *d*(*e*) > 0 denoting the delay on *e*, the *minimum delay many-to-many multicast tree under a fixed TeST topology problem *(MDMP) aims to find an ordering (*v*_1_,…,*v*_*m*−*k*_)*, *v*_*i*_ ∈ *V*∖*S* with a minimum delay.

**Problem 3**

Given an INPUT with every edge *e* ∈ *E* having a weight 0 < *p*(*e*) < 1 representing the working probability on *e*, the *maximum reliability many-to-many multicast tree under a fixed TeST topology problem *(MRMP) aims to find an ordering (*v*_1_,…, *v*_*m*−*k*_)*, *v*_*i*_ ∈ *V*∖*S* with a maximum reliability.

### 3.3. Euclidean Graphs

Given an undirected graph *G* = (*V*, *E*, *ω*) with every edge *e* ∈ *E* having a weight *ω*(*e*), we construct two types of *Euclidean graphs* based on *G* in the following. 

#### 3.3.1. Shortest-Path Graph

When *ω*(*e*), ∀*e* ∈ *E* in *G* = (*V*, *E*, *ω*) represents the cost *c*(*e*) or delay *d*(*e*) on *e*, the path between *v*_*i*_ and *v*_*j*_ with a minimum weight (e.g., cost or delay) is called a *shortest path* (SP), denoted as π*[*v*_*i*_, *v*_*j*_]. So,

(17) $$\begin{array}{c} \omega \left(\pi^{*}\left[v_{i},v_{j}\right]\right)=\underset{\pi [v_{i},v_{j}]}{\min}\;\;\sum_{e\in \pi [v_{i},v_{j}]}^{}\omega \left(e\right). \end{array}$$

All-pairs SPs in *G* form a set, denoted as Σ, that is,

(18) $$\begin{array}{c} \Sigma =\left\{\pi^{*}\left[v_{i},v_{j}\right]:\forall v_{i},\;v_{j}\in V,v_{i}\neq v_{j}\right\}. \end{array}$$

Next we construct a new graph Σ(*G*) = (*V*, Σ, *ω*) from *G* such that the edge between *v*_*i*_ and *v*_*j*_ just represents an SP π*[*v*_*i*_, *v*_*j*_] in *G*. We call Σ(*G*) as a *Shortest-Path Graph* (SPG). Evidently, Σ(*G*) is a complete graph. 

#### 3.3.2. Maximum-Reliability-Path Graph

When *ω*(*e*), ∀*e* ∈ *E* in *G* = (*V*, *E*, *ω*) represents the working probability *p*(*e*) on *e*, the path between *v*_*i*_ and *v*_*j*_ with a maximum working probability is called a *maximum reliability path* (MRP), denoted as π*[*v*_*i*_, *v*_*j*_]. So,

(19) $$\begin{array}{c} p\left(\pi^{*}\left[v_{i},v_{j}\right]\right)=\underset{\pi [v_{i},v_{j}]}{\max}\;\prod_{e\in \pi [v_{i},v_{j}]}p\left(e\right). \end{array}$$

All-pairs MRPs in *G* form a set, denoted as Π, that is,

(20) $$\begin{array}{c} \Pi =\left\{\pi^{*}\left[v_{i},v_{j}\right]:\forall v_{i},\;v_{j}\in V,\;v_{i}\neq v_{j}\right\}. \end{array}$$

Next, we construct a complete graph Π(*G*) = (*V*, Π, *p*) from *G* such that the edge between *v*_*i*_ and *v*_*j*_ just represents an MRP π*[*v*_*i*_, *v*_*j*_]. We call Π(*G*) a *Maximum- Reliability-Path Graph* (MRPG). 

## 4. Minimum Cost Many-to-Many Multicast Tree under a Fixed Topology

In this section, we study the centralized and decentralized MCMP, respectively. 

### 4.1. Centralized MCMP

According to discussions above in Section 2, the essence of finding a minimum cost multicast tree under a given TeST in the centralized MCMP is to find a minimum cost realization of the rooted TeST topology. In this subsection, we devise a polynomial-time exact algorithm for the centralized MCMP.

Let *R*(*T*_*u*_) be a realization of *T*_*u*_ with *u* mapped to *v* in the centralized MCMP. Let *C*[*u*][*v*] denote the cost of *R*(*T*_*u*_). When *u* ∈ *U* is a leaf of *T*, considering that *T*_*u*_ contains a single node, we set *C*[*u*][*u*] = 1. Otherwise, for every $u\in U\setminus \bar{S}$, we can use (21) to compute *C*[*u*][*v*] for all $v\in V\setminus \bar{S}$,

(21) $$\begin{array}{c} C\left[u\right]\left[v\right]=\sum_{u_{k}\in \Delta \left(u\right)}^{}\;\underset{v_{k}\in V\setminus \bar{S}}{\min}\left\{C\left[u_{k}\right]\left[v_{k}\right]+c\left(v_{k},v\right)\right\}, \end{array}$$

where *c*(*v*_*k*_, *v*) denotes the cost of π*[*v*_*k*_, *v*]. We use (21) to compute all of *C*[*u*][*v*] by the dynamic programming method until *C*[*r*][*r*] is obtained, and then we can trace out an exact solution of the centralized MRMP easily if some bookkeeping information is saved during the computation.

Above analysis leads to algorithm MCCT that can find a minimum cost centralized multicast tree under a TeST, which is denoted as (*v*_1_,…,*v*_*α*−*λ*_)_C_*, in a polynomial time, see Theorem 1. We can use the approach in [21] to implement algorithm MCCT. So as to compute all *C*[*u*][*v*], we need to get all-pairs shortest paths in *G* beforehand, namely, Σ_*c*_(*G*) = (*V*, Σ_*c*_, *c*) where Σ_*c*_ denotes (18) with a cost on each edge of *G*. For the purpose, we can use the Floyd's algorithm, *n* times Dijkstra's algorithm, and so forth.

**Theorem 1**

Given an INPUT as *I*, algorithm MCCT can find an optimal solution of the centralized MCMP correctly in *O*(*n* ^3^ + *β*(*n*−*λ*)^2^) time.

**Proof**

Step_1 takes *O*(*n* ^3^) time to find all-pairs shortest paths by using the Floyd's algorithm. Step_2 first spends *O*(*αn*) time to initialize *C*[*u*][*v*] for all *u* ∈ *U* and *v* ∈ *V*, and then uses (21) to compute *C*[*u*][*v*] for all ∈*U*∖*S*, whose time complexity is at most

(22) O(|Δ(r)|·|V∖S¯|)+∑u∈U∖S¯ ∑v∈V∖S¯O(|Δ(u)|·|V∖S¯|) =O(|Δ(r)|·|V∖S¯|)+O(∑u∈U∖S¯|Δ(u)|·∑v∈V∖S¯|V∖S¯|) =O(|Δ(r)|·|V∖S¯|)+O(|V∖S¯|2·∑u∈U∖S¯|Δ(u)|) ≤O(|V∖S¯|2·∑u∈U∖S|Δ(u)|) =(1)O(|F|(n−λ−1)2) =O(β(n−λ)2).

Step_3 only takes *O*(*α* − *λ*) time if we save some book-keepings information during the computation in Step_2.

### 4.2. Decentralized MCMP

By the discussions above in Section 2, to find a minimum cost multicast tree under a given TeST in the decentralized MCMP is essentially to find a minimum cost realization of the unrooted TeST topology. Since an unrooted TeST can be transformed into a rooted TeST by assigning it any nonleaf as its root, we can adapt the algorithm MCCT for finding a minimum cost decentralized multicast tree under an unrooted TeST, which is denoted as (*v*_1_,…, *v*_*α*−*λ*_)_D_*. The resultant algorithm is named as MCDT. The main differences between MCDT and MCCT are that $v_{k}\in V\setminus \bar{S}$ in (21) is changed to *v*_*k*_ ∈ *V*∖*S* in

(23) $$\begin{array}{c} C\left[u\right]\left[v\right]=\sum_{u_{k}\in \Delta \left(u\right)}^{}\;\underset{v_{k}\in V\setminus S}{\min}\left\{C\left[u_{k}\right]\left[v_{k}\right]+c\left(v_{k},v\right)\right\}, \end{array}$$

and MCDT removes the restriction of *r* mapped to *r* from MCCT. Based on Theorem 1., we calculate the time complexity of algorithm MCDT, shown in Theorem 2.

**Theorem 2**

Given an INPUT as *I*, algorithm MCDT can find an optimal solution of the decentralized MCMP correctly in *O*(*n* ^3^ + *β*(*n* − *λ*)^2^) time.

**Proof**

It is similar to the proof of Theorem 1.

## 5. Minimum Delay Many-to-Many Multicast Tree under a Fixed Topology

In this section, we study the centralized and decentralized MDMP, respectively. 

### 5.1. Centralized MDMP

In the centralized MDMP, to find a minimum delay multicast tree under a given TeST topology is to find a minimum delay realization of the rooted TeST. In this subsection, we design a polynomial-time exact algorithm for the centralized MDMP. 

Let *R*(*T*_*u*_) be a realization of *T*_*u*_ with *u* mapped to *v* in the centralized MDMP. The delay of *R*(*T*_*u*_) is equal to the radius of *R*(*T*_*u*_). We let *Y*[*u*][*v*] denote the radius of *R*(*T*_*u*_). When *u* ∈ *U* is a leaf of *T*, we set *Y*[*u*][*u*] = 0. Otherwise, for each $u\in U\setminus \bar{S}$, we can use (24) to compute *Y*[*u*][*v*] for all $v\in V\setminus \bar{S}$,

(24) $$\begin{array}{c} Y\left[u\right]\left[v\right]=\underset{u_{k}\in \Delta \left(u\right)}{\max}\;\underset{v_{k}\in V\setminus \bar{S}}{\min}\left\{Y\left[u_{k}\right]\left[v_{k}\right]+d\left(v_{k},v\right)\right\}, \end{array}$$

where *d*(*v*_*k*_, *v*) denotes the delay of π*[*v*_*k*_, *v*]. We use (24) to compute *Y*[*u*][*v*] recursively until *Y*[*r*][*r*] is achieved, and then we can trace out an exact solution of the centralized MDMP easily if some bookkeeping information is saved during the whole computation.

Based on above analysis, we devise algorithm MDCT to find a minimum delay centralized multicast tree under a TeST, which is denoted as (*v*_1_,…,*v*_*α*−*λ*_)_C_^#^, in a polynomial time; see Theorem 3. We also can use the way in [21] to execute algorithm MDCT. In order to compute all *Y*[*u*][*v*], we need to get all-pairs shortest paths in *G* beforehand, namely, Σ_*d*_(*G*) = (*V*, Σ_*d*_, *d*) in which Σ_*d*_ denotes (18) with a delay on every edge of *G*. Here, we can use the Floyd's algorithm to get Σ_*d*_(*G*).

**Theorem 3**

Given an INPUT as *I*, algorithm MDCT can find an optimal solution of the centralized MDMP correctly in *O*(*n* ^3^ + *β*(*n* − *λ*)^2^) time.

**Proof**

It is similar to the proof of Theorem 1.

### 5.2. Decentralized MDMP

The essence of finding a minimum delay multicast tree under a given TeST in the decentralized MDMP is to find a minimum delay realization of the unrooted TeST topology. 

First of all, we can always use the method in [22] to transform an unrooted tree into a rooted tree and further into a binary tree, denoted as *T* ^B^ = (*U* ^B^, *F* ^B^). Clearly, |*U* ^B^∖*S*| = |*S* | −1 = *λ* − 1. For any nonleaf *u* ∈ *U* ^B^∖*S*, let *u*_*l*_ and *u*_*r*_ denote its left and right child, respectively. Let *R*(*T*_*u*_^B^) be a realization of *T*_*u*_^B^ with *u* mapped to *v* in the decentralized MDMP. The delay of *R*(*T*_*u*_^B^) is equal to the diameter of *R*(*T*_*u*_^B^) and denoted by *X*[*u*][*v*]. When *u* ∈ *U* ^B^ is a leaf of *T*_*u*_^B^, we set *X*[*u*][*u*] = 0 since *T*_*u*_^B^ has a single node. Otherwise, for each *u* ∈ *U* ^B^∖*S*, we can use (25) to compute *X*[*u*][*v*] for all *v* ∈ *V*∖*S*,

(25) X[u][v]=min⁡vl,vr∈V∖Smax⁡⁡{X[ul][vl],X[ur][vr],     Y[ul][vl]+Y[ur][vr]+d(vl,v)+d(vr,v)},

where *u*_*l*_ is mapped to *v*_*l*_ and *u*_*r*_ is mapped to *v*_*r*_, *d*(*v*_*l*_, *v*) and *d*(*v*_*r*_, *v*) denote the delay of π*[*v*_*l*_, *v*] and π*[*v*_*r*_, *v*], respectively, and both of *Y*[*u*_*l*_][*v*_*l*_], *Y*[*u*_*r*_][*v*_*r*_] can be derived from

(26) $$\begin{array}{c} Y\left[u\right]\left[v\right]=\max\left\{\begin{array}{c} \underset{v_{l}\in V\setminus S}{\min}\left\{Y\left[u_{l}\right]\left[v_{l}\right]+d\left(v_{l},v\right)\right\}\\ \;\underset{v_{r}\in V\setminus S}{\min}\left\{Y\left[u_{r}\right]\left[v_{r}\right]+d\left(v_{r},v\right)\right\} \end{array}\right\}. \end{array}$$

We can use (25) to compute *X*[*u*][*v*] recursively, and then we can trace out an exact solution of the decentralized MDMP, denoted as (*v*_1_,…,*v*_*α*−*λ*_)_D_^#^, if some bookkeeping information are is during the whole computation. The resulting algorithm is called MDDT, whose time complexity is presented in Theorem 4.

**Theorem 4**

Given an INPUT as *I*, algorithm MDDT can find an optimal solution of the decentralized MDMP correctly in *O*(*n* ^3^ + *λ*(*n* − *λ*)^3^) time.

**Proof**

It is similar to the proofs of Theorems 1 and 2. Step_2 takes *O*((2*λ* − 1)*n*) time to initialize *X*[*u*][*v*] and *Y*[*u*][*v*] for all *u* ∈ *U* ^B^ and *v* ∈ *V* and then computes *X*[*u*][*v*] and *Y*[*u*][*v*] for all *u* ∈ *U* ^B^∖*S* and *v* ∈ *V*∖*S*, whose time complexity is at most

(27) ∑u∈UB∖S ∑v∈V∖S(O(|V∖S|)+O(|V∖S|2))  =O(|UB∖S|·|V∖S|3)  =O(λ(n−λ)3).

Therefore the time complexity of algorithm MDDT is no more than *O*(*n* ^3^ + *λ*(*n* − *λ*)^3^).

## 6. Maximum Reliability Many-to-Many Multicast Tree under a Fixed Topology

In this section, we study the centralized and decentralized MRMP, respectively. 

### 6.1. Constructing an MRPG

MRPG based on *G* will play an important role in the design of algorithm for MRMP in *G*. According to the discussions in Section 3, MRPG based on *G* is an Euclidean graph comprising all-pairs MRPs in *G*. So the key work of constructing MRPG is to devise an efficient algorithm for finding all-pairs MRPs. In this section, we present such an algorithm with a cubic time.

Firstly, we introduce a fundamental Lemma 5.

**Lemma 5**

Given an undirected graph *G* = (*V*, *E*, *p*) with every edge *e* ∈ *E* having an independent working probability, for any path *π*[*v*_*i*_, *v*_*j*_] composed of two subpaths *π*[*v*_*i*_, *v*_*k*_] and *π*[*v*_*k*_, *v*_*j*_], *π*[*v*_*i*_, *v*_*j*_] is an MRP in *G* if and only if both *π*[*v*_*i*_, *v*_*k*_] and *π*[*v*_*k*_, *v*_*j*_] are MRPs in *G*.

**Proof**

On one hand, if *π*[*v*_*i*_, *v*_*j*_] is an MRP, we can verify that the combination of *π*′[*v*_*i*_, *v*_*k*_] and *π*[*v*_*k*_, *v*_*j*_] forms a more reliable path than *π*[*v*_*i*_, *v*_*j*_] provided that *π*′[*v*_*i*_, *v*_*k*_] is a more reliable path than *π*[*v*_*i*_, *v*_*k*_] or the combination of *π*[*v*_*i*_, *v*_*k*_] and *π*′[*v*_*k*_, *v*_*j*_] forms a more reliable path than *π*[*v*_*i*_, *v*_*j*_] provided that *π*′[*v*_*k*_, *v*_*j*_] is a more reliable path than *π*[*v*_*k*_, *v*_*j*_]. This causes a contradiction. On the other hand, if *π*[*v*_*i*_, *v*_*k*_] and *π*[*v*_*k*_, *v*_*j*_] are both MRP's, we can verify that either *π*′[*v*_*i*_, *v*_*k*_] is more reliable than *π*[*v*_*i*_, *v*_*k*_] or *π*′[*v*_*k*_, *v*_*j*_] is more reliable than *π*[*v*_*k*_, *v*_*j*_] provided that *π*′[*v*_*i*_, *v*_*j*_] consisting of *π*′[*v*_*i*_, *v*_*k*_] and *π*′[*v*_*k*_, *v*_*j*_] is a more reliable path than *π*[*v*_*i*_, *v*_*j*_]. This causes a contradiction.

From Lemma 5, we claim that the most reliable paths in *G* satisfy the* triangle inequality*, based on which we can design a dynamic programming algorithm for finding all- pairs MRP's. 

For any edge *e* = {*v*_*i*_, *v*_*j*_} ∈ *E*, we rewrite *p*(*e*) to be *p*(*v*_*i*_, *v*_*j*_). We can use (28) to construct a probability matrix *P* = (*P*_*i*,*j*_)_*n*×*n*_,

(28) $$\begin{array}{c} P_{i,j}=\{\begin{array}{cc} 1 & \text{if}\;i=j,\\ p\left(v_{i},v_{j}\right) & \text{if}\;i\neq j,\left\{v_{i},v_{j}\right\}\in E,\\ 0 & \text{if}\;i\neq j,\left\{v_{i},v_{j}\right\}\notin E. \end{array} \end{array}$$

We use *p* ^(*k*)^(*i*, *j*) to denote the working probability of a current MRP between *v*_*i*_ and *v*_*j*_ after *v*_*k*_ is introduced. We set *p* ^(0)^(*i*, *j*) = *P*_*i*,*j*_ initially and then use (29) to compute *p* ^(*k*)^(*i*, *j*) for *k* from 1 to *n*.

(29) p(k)(i,j)=max⁡⁡{p(k−1)(i,j),    p(k−1)(i,k)×p(k−1)(k,j)}.

Finally, *p* ^(*n*)^(*i*, *j*) is the working probability of the MRP in *G* between *v*_*i*_ and *v*_*j*_. 

In essence, above idea of using (29) recursively forms our dynamic programming algorithm for finding all-pairs MRP's, namely, constructing Π(*G*) = (*V*, Π, *p*), which is described as procedure CMRP (Procedure 1). The time complexity of CMRP is shown in Lemma 6.

**Lemma 6**

Given an undirected edge-weighted graph *G* = (*V*, *E*, *p*) with *n* nodes and *m* edges in which every edge has an independent working probability, procedure CMRP can find all-pairs maximum reliability paths in *O*(*n* ^3^) time.

**Proof**

Step_1 spends *O*(*n* ^2^) time to initialize *p* ^(0)^(*i*, *j*) for *i* = 1,2,…, *n* and *j* = 1,2,…, *n*. For each *k* = 1,2,…, *n*, Step_2 spends *O*(*n* ^2^) time to compute *p* ^(*k*)^(*i*, *j*) for *i* = 1,…, *n* and *j* = 1,…, *n*. Therefore, the time complexity of CMRP is *O*(*n* ^3^).

### 6.2. Algorithms

In this section, we present an exact algorithm for the centralized and decentralized MRMP, respectively. 

#### 6.2.1. Centralized MRMP

The essence of finding a maximum reliability multicast tree under a TeST topology in the centralized MRMP is to find a maximum reliability realization of the rooted TeST.

Let *R*(*T*_*u*_) be a realization of *T*_*u*_ with *u* mapped to *v* in the centralized MRMP. The reliability of *R*(*T*_*u*_) refers to its radius reliability, which is denoted as *φ*[*u*][*v*]. When *u* ∈ *U* is a leaf of *T*, we set *φ*[*u*][*u*] = 0. Otherwise, for each $u\in U\setminus \bar{S}$, we can use (30) to compute *φ*[*u*][*v*] for all $v\in V\setminus \bar{S}$,

(30) $$\begin{array}{c} \varphi \left[u\right]\left[v\right]=\underset{u_{k}\in \Delta \left(u\right)}{\min}\;\underset{v_{k}\in V\setminus \bar{S}}{\max}\left\{\varphi \left[u_{k}\right]\left[v_{k}\right]\times p\left(v_{k},v\right)\right\}, \end{array}$$

where *p*(*v*_*k*_, *v*) denotes the reliability of π*[*v*_*k*_, *v*]. We use (30) to compute *φ*[*u*][*v*] recursively until *φ*[*r*][*r*] is got, and then we can trace out an exact solution of the centralized MRMP if some bookkeeping information is saved during the whole computation.

Above analysis can be described as algorithm MRCT. It can find a maximum reliability centralized multicast tree under a TeST, denoted as   (*v*_1_,…, *v*_*α*−*λ*_)_C_^△^, in a polynomial time; see Theorem 7. We use the method in [21] to perform algorithm MRCT. In order to compute *φ*[*u*][*v*], we need to get all-pairs maximum reliability paths in *G* beforehand, namely, Π(*G*) = (*V*, Π, *p*). This work can be accomplished by procedure CMRP.

**Theorem 7**

Given an INPUT as *I*, algorithm MRCT can find an optimal solution of the centralized MRMP correctly in *O*(*n* ^3^ + *β*(*n* − *λ*)^2^) time.

**Proof**

It is similar to the proof of Theorem 1.

#### 6.2.2. Decentralized MRMP

To find a maximum reliability multicast tree under a TeST in the decentralized MDMP is essentially to find a maximum reliability realization of the unrooted TeST. We can use the way in [22] to transform an unrooted tree into a rooted tree and further into a binary tree *T* ^B^ = (*U* ^B^, *F* ^B^). And some definitions and notations therein are still used here. Let *R*(*T*_*u*_^B^) be a realization of *T*_*u*_^B^ with *u* mapped to *v* in the decentralized MRMP. The reliability of *R*(*T*_*u*_^B^) refers to its diameter reliability, which is denoted as *ψ*[*u*][*v*]. When *u* ∈ *U* ^B^ is a leaf of *T*_*u*_^B^, we set *ψ*[*u*][*u*] = 0. Otherwise, for each *u* ∈ *U* ^B^∖*S*, we can use (31) to compute *ψ*[*u*][*v*] for all *v* ∈ *V*∖*S*,

(31) ψ[u][v]=max⁡vl,vr∈V∖Smin⁡⁡{ψ[ul][vl],ψ[ur][vr],     φ[ul][vl]×φ[ur][vr]×p(vl,v)×p(vr,v)},

where *u*_*l*_ is mapped to *v*_*l*_ and *u*_*r*_ is mapped to *v*_*r*_, *p*(*v*_*l*_, *v*) and *p*(*v*_*r*_, *v*) denote the reliability of π*[*v*_*l*_, *v*] and π*[*v*_*r*_, *v*], respectively, and both of *φ*[*u*_*l*_][*v*_*l*_], *φ*[*u*_*r*_][*v*_*r*_] can be obtained by using

(32) $$\begin{array}{c} \varphi \left[u\right]\left[v\right]=\min\left\{\begin{array}{c} \underset{v_{l}\in V\setminus S}{\max}\left\{\varphi \left[u_{l}\right]\left[v_{l}\right]\times p\left(v_{l},v\right)\right\}\\ \;\underset{v_{r}\in V\setminus S}{\max}\left\{\varphi \left[u_{r}\right]\left[v_{r}\right]\times p\left(v_{r},v\right)\right\} \end{array}\right\}. \end{array}$$

We can use (31) to compute *ψ*[*u*][*v*] recursively, and then we can trace out an exact solution of the decentralized MRMP, denoted as   (*v*_1_,…,*v*_*α*−*λ*_)_D_^△^, if some bookkeeping information is saved during the whole computation. This leads to algorithm MRDT, whose time complexity is shown in Theorem 8.

**Theorem 8**

Given an INPUT as *I*, algorithm MRDT can find an optimal solution of the decentralized MRMP correctly in *O*(*n* ^3^ + *λ*(*n* − *λ*)^3^) time.

**Proof**

It is similar to the proof of Theorem 4.

## 7. Illustrative Examples

In this section, we take the network and the binary tree topology shown in Figure 1 as an example to illustrate the six algorithms proposed above (Algorithms 1–6). 

**Figure 2:**
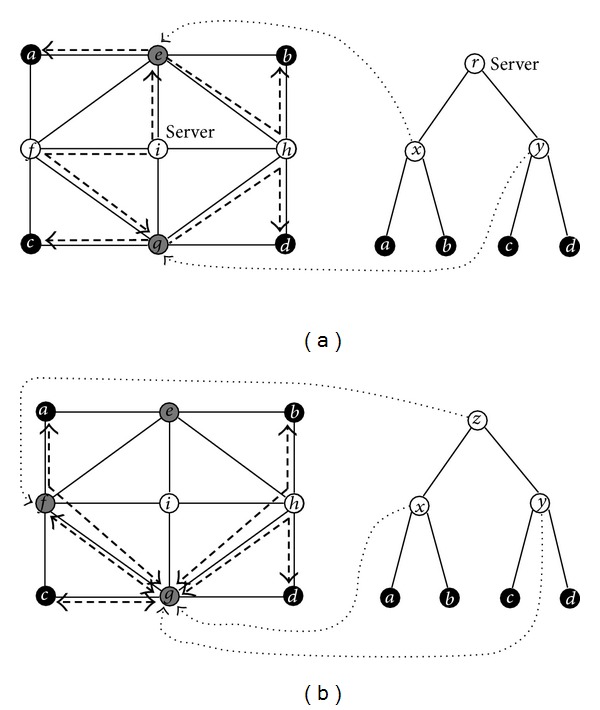
For ease of view, we neglect the numbers on edges. The example network for MCMP and the binary tree topology are both the same as those shown in Figure 1 and the integer on every edge represents its quantity of cost. The minimum cost multicast tree for the centralized MCMP is drawn with the bold dashed edges on the top subfigure and the one for the decentralized MCMP is drawn on the bottom subfigure.

Suppose the integer on every link of the network in Figure 1 represents the quantity of cost on the link. For instance, each unit of cost is 0.8 dollar; then the cost on edge {*b*, *e*} is 3.2 dollars. We apply algorithm MCCT to solve the centralized MCMP. MCCT first uses the Floyd's algorithm to find all-pairs minimum cost paths in the given network, that is, constructing the SPG Σ_*c*_(*G*) = (*V*, Σ_*c*_, *c*) with respect to the link costs of network then computes the values of (21) recursively, and finally terminates with an optimal ordering (*e*, *g*, *i*) of the centralized MCMP. So we know that *x*, *y*, *r* are mapped to *e*, *g*, *i*, respectively, and then derive an optimal solution from Σ_*c*_(*G*) distinguished by bold dashed edges on the top subfigure of Figure 2; for example, the communication between *x* and *b* is established by the minimum cost path *e* -*h* - *b* and the communication between *r* and *y* is established by the minimum cost path *i*-*f*-*g*. Similarly we can apply algorithm MCDT to solve the decentralized MCMP. MCDT first constructs Σ_*c*_(*G*), then computes the values of (23) recursively, and finally ends with (*g*, *g*, *f*). Hence we can derive an optimal solution to the decentralized MCMP from Σ_*c*_(*G*), distinguished by bold dashed edges on the bottom subfigure of Figure 2. 

**Figure 3:**
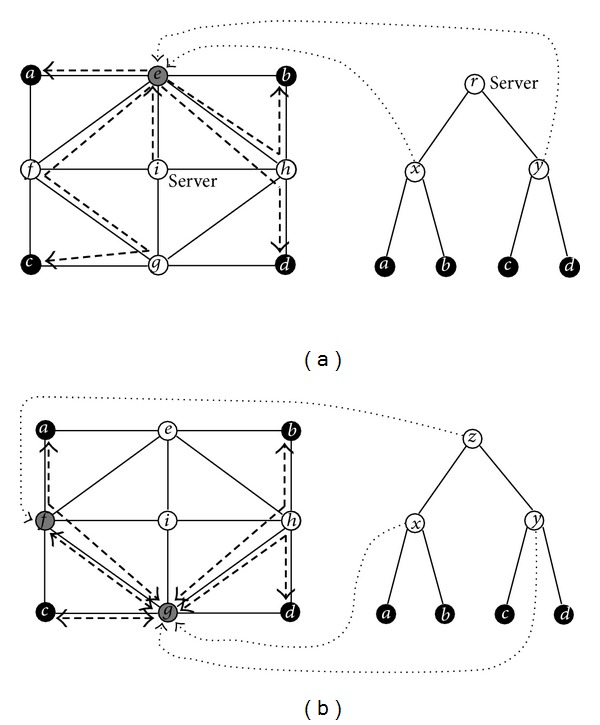
The example network for MDMP and the binary tree topology are both the same as those shown in Figure 1 and the integer on every edge represents its amount of delay. The minimum delay multicast tree for the centralized MDMP is drawn with the bold dashed edges on the top subfigure and the one for the decentralized MDMP is drawn on the bottom subfigure.

Suppose the integer on every link of the network in Figure 1 represents its amount of delay. For example, every unit of delay is 1 ms, then the delay on link {*e*, *f*} is 3 ms. We apply algorithm MDCT to solve the centralized MDMP. MDCT first uses the Floyd's algorithm to find all-pairs minimum delay paths in the given network, that is, constructing the SPG Σ_*d*_(*G*) = (*V*, Σ_*d*_, *d*) with respect to the link delays of network, then computes the values of (24) recursively, finally ends with an optimal ordering (*e*, *e*, *i*) of the centralized MDMP. So we know that *x*, *y*, *r* are mapped to *e*, *e*, *i*, respectively, then derive an optimal solution from Σ_*d*_(*G*) distinguished by dashed edges on the top subfigure of Figure 3. Similarly, we apply MDDT to the decentralized MDMP. MDDT first constructs Σ_*d*_(*G*), and then computes the values of (25) and (26) recursively, finally terminates with (*g*, *g*, *f*). We can derive an optimal solution to the decentralized MDMP from Σ_*d*_(*G*), which is distinguished by bold dashed edges on the bottom subfigure of Figure 3 and equal to the optimal solution to the decentralized MCMP.

**Figure 4:**
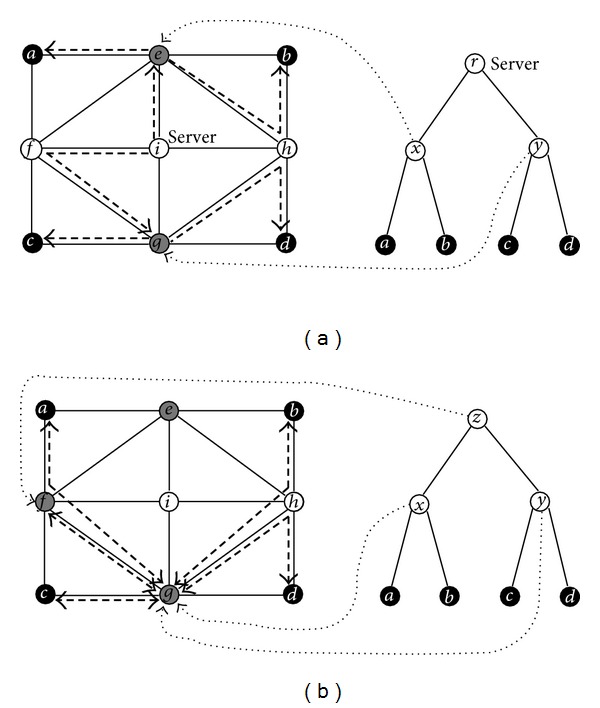
The example network for MRMP and the binary tree topology are both the same as those shown in Figure 1 and the integer on every edge represents the parameter of the linear probability function. The maximum reliability multicast tree for the centralized MRMP is drawn with the bold dashed edges on the top subfigure and the one for the decentralized MRMP is drawn on the bottom subfigure.

Suppose that every link *e* ∈ *E* of the network given in Figure 1 has an independent working probability whose value is a linear function in the integer *δ*(*e*) on *e*, formulated as *p*(*e*) = 1 − 0.01 × *δ*(*e*). For instance, the working probability of {*a*, *e*} is 0.98. We apply algorithm MRCT to solve the centralized MRMP. MRCT first uses procedure CMRP to find all-pairs maximum reliability paths in the network, that is, constructing the MRPG Π(*G*) = (*V*, Π, *p*) with respect to the link probabilities of the given network, then computes the values of (30) recursively, finally terminates with an optimal ordering (*e*, *g*, *i*) of the centralized MRMP. We can get an optimal solution from Π(*G*) distinguished by bold dashed edges on the top subfigure of Figure 4. Similarly, we can apply algorithm MRDT to solve the decentralized MRMP. MRDT first constructs Π(*G*), then computes the values of (31) and (32) recursively, and finally ends with (*g*, *g*, *f*). Hence we can derive an optimal solution to the decentralized MRMP from Π(*G*), distinguished by bold dashed edges on the bottom subfigure of Figure 4. Evidently, the optimal solution to the centralized MRMP is same as that to the centralized MCMP as well as the optimal solution to the decentralized MRMP is same as that to the decentralized MCMP.

## 8. Conclusions

This paper introduces the architecture of a many-to-many multicast tree with fixed topology, reduces it to a realization of the given TeST topology, and applies the idea of under a fixed topology to deal with three optimization problems, that is, the minimum cost, minimum delay, and maximum reliability multicast tree under a fixed topology problem. Each problem includes the centralized and decentralized versions. For the both versions of each problem, an exact algorithm is devised using the dynamic programming approach, respectively. On the condition that we are given a collection of alternative tree topologies, it is of interests to explore a best topology from all the alternative tree topologies. 

Moreover, if we consider two or more weights on every link of a network, it is an interesting and important research topic how to devise an efficient algorithm for the multicast tree problem under a fixed topology with multiple objectives or with a single objective and at least one constraint. 

## Figures and Tables

**Algorithm 1:**
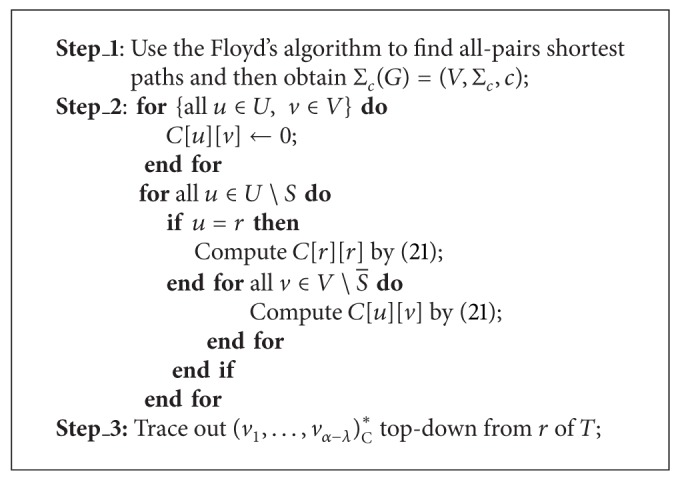
(*v*_1_,…, *v*_*α*−*λ*_)_C_* = MCCT[*I*].

**Algorithm 2:**
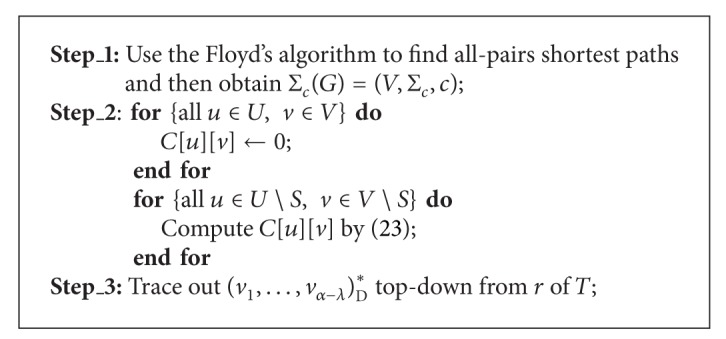
(*v*_1_,…, *v*_*α*−*λ*_)_D_* = MCDT[*I*].

**Algorithm 3:**
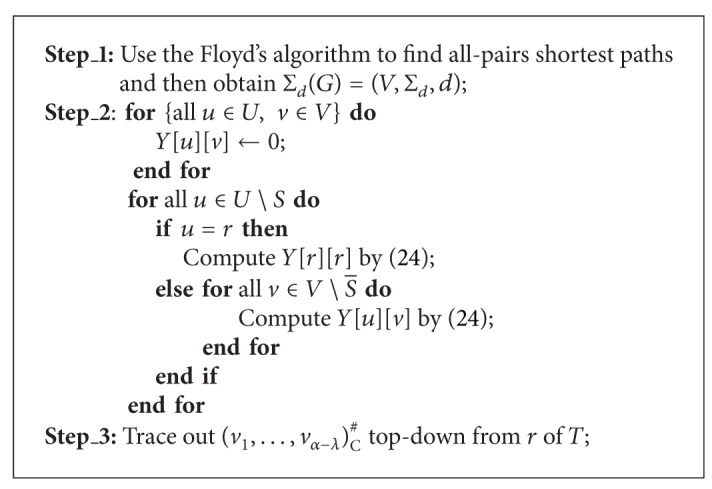
(*v*_1_,…, *v*_*α*−*λ*_)_C_^#^ = MDCT[*I*].

**Algorithm 4:**
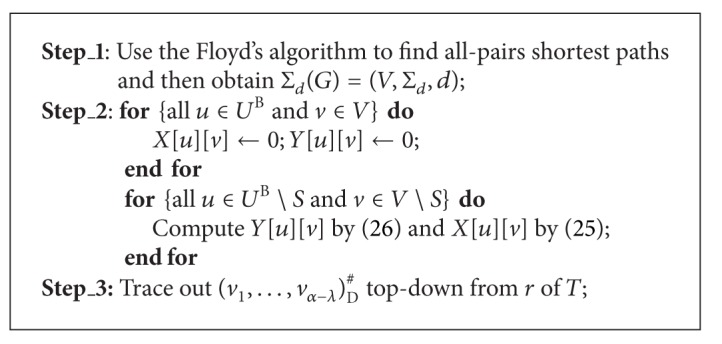
(*v*_1_,…, *v*_*α*−*λ*_)_D_^#^ = MDDT[*I*].

**Procedure 1:**
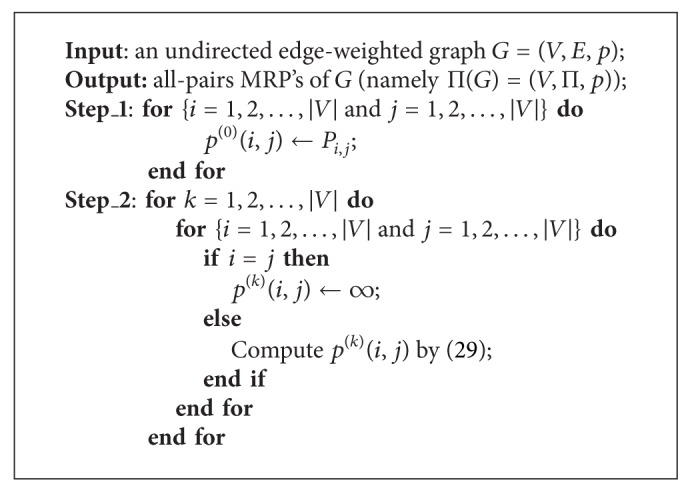
Procedure CMRP.

**Algorithm 5:**
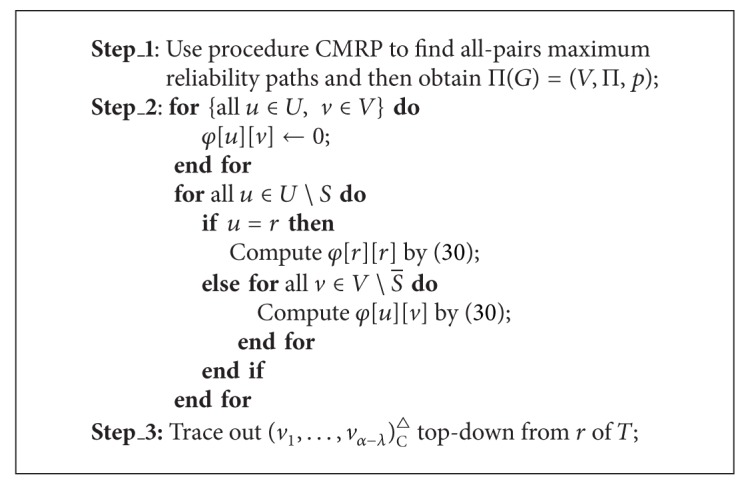
(*v*_1_,…, *v*_*α*−*λ*_)_C_^△^ = MRCT[*I*].

**Algorithm 6:**
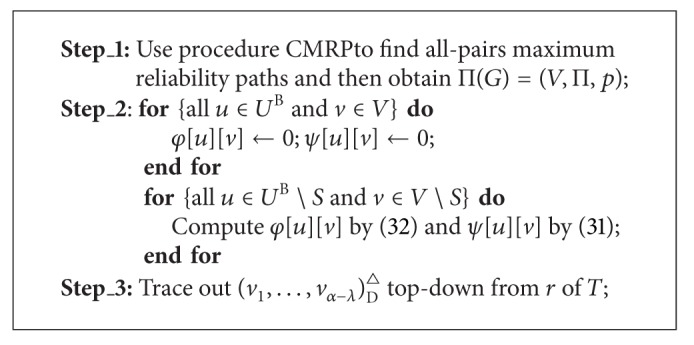
(*v*_1_,…, *v*_*α*−*λ*_)_D_^△^ = MRDT[*I*].
